# Computational tools for genomic data de-identification: facilitating data protection law compliance

**DOI:** 10.1038/s41467-021-27219-2

**Published:** 2021-11-29

**Authors:** Alexander Bernier, Hanshi Liu, Bartha Maria Knoppers

**Affiliations:** Centre of Genomics and Policy, McGill University, Faculty of Medicine, 740, avenue Dr. Penfield, suite 5200, Montreal, QC H3A 0G1 Canada

**Keywords:** Molecular medicine, Data publication and archiving

## Abstract

In this opinion piece, we discuss why computational tools to limit the identifiability of genomic data are a promising avenue for privacy-preservation and legal compliance. Even where these technologies do not eliminate all residual risk of individual identification, the law may still consider such data anonymised.

## The intersection of scientific enterprise and data protection law

Legislators worldwide have implemented data protection laws governing how organisations and institutions can use identifiable personal data^1^. The specifics of such laws differ from one country to another. Common features, however, include the requirement to establish a legal justification before using personal data, and to implement organisational and technical measures to hold the data secure. The core feature of data protection legislation is the presence of a number of foundational principles. These principles, for example, require institutions using data to minimise their collection of data, restrict the use of such data to the purposes established at the moment of data collection, and to eliminate personal data that are no longer needed^2^.

Entities that use personal data can also be required to perform risk assessments prior to and during data use, maintain records of data use, and cooperate in government audits if a data breach should occur^3^. These data users are also bound to respect the rights of individuals concerning their personal information. Such rights include the right to access their personal data and the right to request data correction. Penalties for non-compliance are grim^3^.

Data protection legislation has been lauded as a critical milestone in the oversight of large multinational corporations. However, such legislation has proven less apt in the regulation of biomedical data uses in the health sector. Health sector institutions struggle to understand how to best reconcile their activities with the requirements of data protection law^4^. The challenges are manifold. First, healthcare institutions often do not have access to the legal expertise required to ensure data protection compliance. Second, biomedical research initiatives, such as research consortia, are often distributed throughout multiple economic sectors and multiple countries, which can require compliance with multiple laws at the same time. Third, biomedical data retained for longitudinal use are often obtained through biobanks of human tissues and through the expenditure of capital and specialised talent. Any requirements to destroy data after a stated period of time are difficult to reconcile with the established principles of biomedical research ethics^5^. Last, the anonymisation of biomedical data so as to withdraw it from the reach of the law creates practical challenges for its continued scientific utility. There is an inherent tension between the data-intensive scientific enterprise and the manipulation of data to reduce its risk of causing individual re-identification^5,6^.

## Genetic information, re-identification risk, and computational tools

Publishing genomic data in open-access repositories raises questions regarding the residual re-identification risk applicable thereto^7^. Re-identification attacks have been attempted that compare limited portions of an individual’s genetic sequence with a reference database of known individuals’ genetic information. The intention of such a re-identification attack is to confirm that the targeted individual matches or does not match the genetic information of one of the comparator individuals^7^.

In response to experimental results demonstrating that a small number of single-nucleotide polymorphisms were sufficient to establish a positive match between a known individual’s genetic information and that same individual’s genetic information held or published in a presumptively anonymised format^8^, efforts have been made to develop technologies that better anonymise genetic information.

Such efforts have led to the creation of technologies offering a good compromise between desirable scientific activities, such as data accessibility, and the interest in robust guarantees of individual anonymity. For example, Beacon systems have been proposed to enable researchers to discover genetic information relevant to their needs, whilst preserving the anonymity of concerned individuals^9,10^.

Beacon systems function as follows. Individual-level genetic information of scientific research interest in potentially identifiable form are held in controlled access databases. This means that a specialised custodian holds the data in a secure database, and provides access to the rich underlying data to accredited researchers who agree to respect applicable governance requirements. However, because the researchers desire to understand whether the available data contains genetic variants or other scientific features that are of research interest to them prior to issuing an application for access to the data, the Beacon system is implemented. This system allows interested parties to ‘query’ the underlying database for the presence or absence of genetic variants that are of scientific interest. Beacon systems have been implemented to increase the utility of biomedical data repositories, in ensuring that researchers can determine that a database holds research data of interest before engaging in the laborious process of requesting and obtaining access to genetic information.

The challenge inherent in genomic Beacons is that some consider these tools to be susceptible to re-identification attacks. Computer scientists have staged re-identification attacks on Beacon systems, in comparing the rare genetic variants of known persons to the genetic variants contained in the Beacon system^11^. This had led to a veritable arms race, with computer scientists iteratively developing more sophisticated re-identification techniques, and subsequent innovators producing methods to defend against the novel risk identified. Proposed methods to safeguard against re-identification attacks involve limiting users to a maximum number of queries. Others are more complex, and return false-negative results once a sufficient number of queries are made targeting genetic information unique to a single genomic record in the underlying database^12^.

Alternate methods of performing data de-identification are tailored to applications in functional genomics, or other circumstances in which genetic data is sequenced, but individual-specific genetic information is not desired. For instance, this is often the case for RNAseq data, which is useful for the purpose of assessing gene expression across different samples or cell types, even if it does not contain any information about genetic variation. In this context, the automated replacement of potentially identifying information with genetic data from an external source, such as a human reference genome, and discarding unmapped sequences, could be a potential de-identification method^13^.

Limitations to both of these methodologies have been expressed in technical literature, either in technical papers responding to such innovations^8,11,12,14^ or directly in the commentaries of their initial creators^12,13^. It is therefore material to consider the promise of technologies for enabling the sharing of biomedical data. It is also relevant to assess the relationship between computational mechanisms for the de-identification of genomic data and data protection law.

## Data protection compliance and genomic de-identification technologies

It is our contention that genomic de-identification technologies are a potent tool for enabling heightened biomedical data sharing and biomedical data use in compliance with data protection law, despite the potential technical limitations thereof.

According to data protection law, de-identification methodologies do not need to reduce the risk of individual re-identification to nil to render the data anonymised. For example, according to the E.U.’s current *General Data Protection Regulation*, data are only considered to be identifiable personal data if the controller, the processor, or a proximate third person has a means of performing individual re-identification at their disposal that is “reasonably likely to be used”^3^. Other jurisdictions also adopt a risk-based approach to assessing whether the data are identifiable personal data. At the moment, Canadian courts consider data to be anonymous unless there is a “serious possibility” of the individual being re-identified, alone or in combination with other available data. In most jurisdictions the threshold for data to be considered anonymised is not ‘zero risk’^15^. Residual risk can still remain in data that are considered anonymised, and indeed certain privacy regulators and health regulators have proposed an acceptable residual risk of an individual being re-identified in a dataset to be in the range of five percent to nine percent. Therefore, technologies such as those described above should still be considered viable methods of producing anonymised data^15^.

We argue that even where methods of genetic data de-identification do not produce anonymised data, these methods remain of high utility for data protection compliance. E.U. data protection legislation requires entities using data to perform ‘data protection by design and by default.’ The computational de-identification of genomic data is a potent tool for discharging this legal requirement. Further, implementing such mechanisms can help satisfy other legal requirements, such as those to perform data minimisation, and to implement context-appropriate security safeguards. Last, computational de-identification methods for genomic data are useful tools for data stewardship.

These methods can be used in combination with traditional organisational controls such as contracts, access policies, and oversight bodies including Scientific Advisory Boards (SABs) and Data Access Committees (DACs)^16^. Consequently, biomedical research consortia and other entities engaged in data sharing exercises could adopt a practice of sharing genomic datasets that have been anonymised in an open or registered access medium, and sharing identifiable datasets in controlled access. This could create a desirable balance between the laudable goal of open science and the need to limit access to data to preserve individual privacy^16^. Governments should stimulate continued research and development in methodologies to anonymise genetic data. This can be achieved using several tools at the disposal of regulators, including research funding, public–private partnerships, and procurement contracts^17^. Legislators should also implement—and continue to revise—specialised legislation further enabling health institutions to use identifiable personal data to perform biomedical research, and so be able to deliver personalised medicine to patients. Indeed, we do not anticipate that data that have been anonymised will be sufficient to meet the growing needs of the burgeoning digital health sector for sufficient volumes of linkable biomedical data to perform statistically significant research.
