# Author Correction: Computational tools for genomic data de-identification: facilitating data protection law compliance

**DOI:** 10.1038/s41467-021-27890-5

**Published:** 2022-01-13

**Authors:** Alexander Bernier, Hanshi Liu, Bartha Maria Knoppers

**Affiliations:** Centre of Genomics and Policy, McGill University, Faculty of Medicine, 740, avenue Dr. Penfield, suite 5200, Montreal, QC H3A 0G1 Canada

**Keywords:** Molecular medicine, Data publication and archiving

Correction to: *Nature Communications* 10.1038/s41467-021-27219-2, published online 29 November 2021.

The original version of this Article listed the wrong reference in the first position in the Reference list. The original reference in the first position read as ‘1. Greenleaf, G. Jamaica adopts a post-GDPR Data Privacy Law. *Priv. Laws Int. Bus. Rep.* **1**, 3–5 (2021).’ The correct reference is ‘1. Greenleaf, G. Global Data Privacy Laws 2021: Despite COVID Delays, 145 Laws Show GDPR Dominance. *Priv. Laws Int. Bus. Rep.* **1**, 3–5 (2021).’

This has been corrected in both the PDF and HTML version of the Article.

